# NORAD orchestrates endometrial cancer progression by sequestering FUBP1 nuclear localization to promote cell apoptosis

**DOI:** 10.1038/s41419-020-2674-y

**Published:** 2020-06-18

**Authors:** Tong Han, Yukang Wu, Xiang Hu, Yaqi Chen, Wenwen Jia, Qizhi He, Yiding Bian, Mengfei Wang, Xudong Guo, Jiuhong Kang, Xiaoping Wan

**Affiliations:** 1 Department of Gynecology, Shanghai First Maternity and Infant Hospital, Tongji University School of Medicine, Shanghai, 200040 China; 2 Clinical and Translational Research Center of Shanghai First Maternity and Infant Hospital, Shanghai Key Laboratory of Signaling and Disease Research, Collaborative Innovation Center for Brain Science, School of Life Sciences and Technology, Institute for Advanced Study, Tongji University, Shanghai, 200092 China; 3 Department of Pathology, Shanghai First Maternity and Infant Hospital, Tongji University School of Medicine, Shanghai, 200040 China

**Keywords:** Endometrial cancer, Apoptosis, Long non-coding RNAs

## Abstract

Long noncoding RNAs (lncRNAs) are emerging as critical regulators in tumor initiation and progression. However, the biological mechanisms and potential clinical application of lncRNA NORAD in endometrial cancer (EC) remain unknown. Herein, we identified NORAD underwent promoter hypermethylation-associated downregulation in EC. Epigenetic inactivation of NORAD was correlated with EC progression (FIGO stage) and poor outcome. Overexpression of NORAD significantly inhibited cell growth and promoted apoptosis in EC cells. Mechanistic studies revealed that multiple regions of NORAD served as a platform for binding with the central domain of anti-apoptotic factor FUBP1. Our findings further indicated that the NORAD/FUBP1 interaction attenuated FUBP1 nuclear localization and thus impaired the occupancies of FUBP1 on its target pro-apoptotic gene promoters, resulting in apoptosis induction in EC. Moreover, knockdown of NORAD promoted tumor growth in the xenograft mice model. While, introduction of NORAD-4 fragment, which bound with FUBP1, successfully reversed tumor growth and apoptosis inhibition mediated by NORAD knockdown in vivo. Our findings provide mechanistic insight into the critical roles of NORAD as a tumor suppressor in EC progression. NORAD could possibly serve as a novel prognostic biomarker and provide the rationale for EC therapy.

## Introduction

Endometrial cancer (EC), originating from the endometrium, is the most common malignant gynecological cancer in women, and its incidence is steadily increasing around the world without improved 5-year survival^1,2^. No acknowledged biomarkers are sensitive and specific enough for diagnosis and prognosis prediction in EC, resulting in the dilemma of risk stratification and further application of adjuvant individualized therapies at early EC stage^2,3^. Therefore, it is of great importance to explore the underlying mechanisms in EC progression.

Emerging evidences support the notion that long noncoding RNAs (lncRNAs), a minimum length of 200 nucleotides, are considered as drivers of multiple cancer phenotypes, including tumor cells sustaining proliferation, viability, motility, and angiogenesis^4–6^. In view of the biological function and specific expression in tumor tissues, lncRNAs are served as biomarkers for tumor diagnosis and therapeutic targets^7,8^. Recent studies found that a highly conserved and abundantly expressed lncRNA, NORAD, could maintain genomic stability by decoying PUMILIO1/2 or binding with RBMX to regulate DNA replication and repair^9–11^. Genome instability was recognized as one of the cancer hallmarks and involved in tumor initiation and progression^12^. Several studies have revealed that NORAD had effects on tumor cell proliferation, apoptosis, and migration via binding with miRNAs or proteins^13^. NORAD was identified as an oncogene in pancreatic and ovarian cancer^14,15^, while its roles in lung and breast cancers have been controversial, indicating a context-dependent role in cancer progression^16^. The function and mechanism of NORAD involved in regulating EC formation and progression remain unexplored.

Far upstream element-binding protein 1 (FUBP1) participated in diverse biological cellular processes as a DNA- and RNA-binding protein^17,18^. Mounting evidences suggested that FUBP1 was upregulated and served as a proto-oncogene in solid cancers^19,20^. FUBP1 repressed p21 mRNA stabilization and regulated pro-apoptotic genes transcription, served as an anti-apoptotic factor in hepatocellular carcinoma^21^. Among gyneocological cancers, FUBP1 was associated with progression-free survival in ovarian cancer^22^. However, there is still an important gap in the understanding of the role of FUBP1 in EC.

Our study demonstrated that NORAD was gradually decreased with the progression of EC due to promoter hypermethylation, and associated with clinical outcome. NORAD could promote EC cell apoptosis in vitro and knockdown of NORAD resulted in tumor malignant growth in vivo. Mechanistically, NORAD bound with FUBP1 and impaired its nuclear localization. Consequently, the NORAD/FUBP1 interaction impeded FUBP1 enrichment on its target gene promoters, resulting in apoptosis induction.

## Results

### NORAD is downregulated in EC due to promoter hypermethylation and correlated with progression and survival of EC patients

**Fig. 1:**
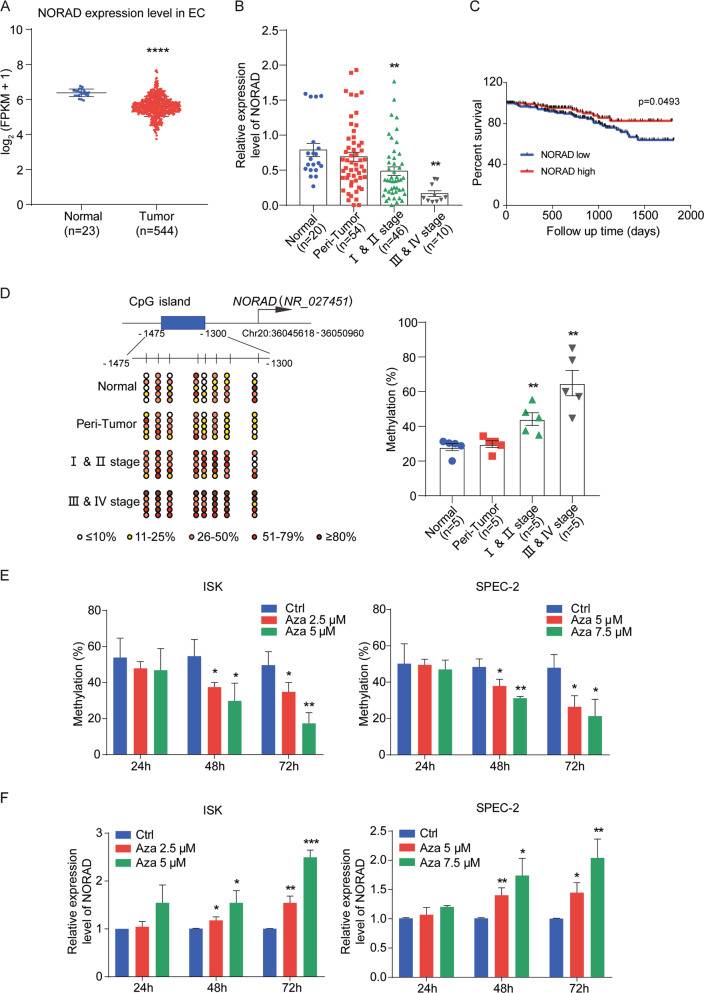
Downregulation of NORAD due to promoter hypermethylation is correlated with progression and prognosis of EC. **a** Relative NORAD expression in the EC patient cohort compared with that in normal endometrial tissues according to TCGA dataset. **b** The NORAD expression level in 20 normal endometrial tissues, 56 EC (including 46 I & II stage and 10 III & IV stage patients), and 54 peri-tumor tissues was detected by qRT-PCR. **c** The survival data from TCGA EC patient cohort containing 308 endometrioid endometrial adenocarcinomas (the major subtype of EC) were analyzed by Kaplan–Meier analysis. **d** Methylation status of the CpG sites at the promoter of NORAD in normal (*n* = 5), peri-tumor (*n* = 5), I & II stage (*n* = 5), and III & IV stage EC patients (*n* = 5) was investigated by bisulfite sequencing. The average percentages of unmethylated and methylated CpGs of 10 clones from each patient were presented by different colors according to the methylated degree. **e** The methylation analysis of NORAD promoter in ISK and SPEC-2 cells with the distinct doses and times of Azacitidine (Aza) treatment, performed by bisulfite sequencing. **f** Restored expression of NORAD after treatment with Aza in EC cells at different doses and times. The results were determined from triplicates, and the error bars represented as the mean ± SEM in patients’ samples, and the mean ± SD in EC cell lines, *P < 0.05, **P < 0.01, ***P < 0.001, ****P < 0.0001. NORAD noncoding RNA activated by DNA damage, EC endometrial cancer, Aza azacitidine.

We first analyzed the RNA-seq data of 544 EC tissues and 23 normal endometrial tissues in The Cancer Genome Atlas (TCGA) and found that the expression level of NORAD was lower in tumor tissues than that in normal tissues (Fig. 1a). We further collected 20 normal endometrial tissues, 54 peri-tumor tissues, and 56 tumor tissues of EC patients and classified into early stage (stage I, II) and advanced stage (stage III, IV) according to Federation of Gynecology and Obstetrics (FIGO) stage. We found that NORAD expression was gradually decreased with the progression of EC compared with that in normal endometrial tissues (Fig. 1b).

In addition, we correlated the NORAD expression level with the clinicopathological characteristics of EC patients via a chi-square test (Table 1). Our results showed that the expression level of NORAD was correlated with FIGO stages and patient age, rather than other clinical factors, such as estrogen receptor (ER) expression, etc. NORAD expression was decreased after 17β-estrogen treatment in ISK (ER-positive) and SPEC-2 (ER-negative) cells in a dose- and time-dependent manner (Supplementary Fig. S1a, b), consistent with no correlation of NORAD and ER expression. To determine the predictive value of NORAD in clinical outcomes, we also evaluated the correlation between NORAD expression and the 5-year overall survival of EC patients, except the patients undergoing hormone therapy or radiation prior surgery. The results illustrated that low NORAD expression predicted a poor prognosis in endometrioid endometrial adenocarcinoma (the major subtype of EC) (Fig. 1c).

**Table 1:** The relation between the expression level of NORAD and clinicopathologic characteristics.

Clinicopathological data	No. of patients	NORAD/GAPDH expression		*χ*^2^	*P*-value
	*n*	Low	High		
Total	56				
*FIGO stage*					
I & II stage	46	20 (43.5%)	26 (56.5%)	4.38	<0.05^*^
III & IV stage	10	8 (80%)	2 (20%)		
*Grade*					
Grade I	36	13 (36.1%)	23 (63.9%)	5.45	>0.05
Grade II	6	5 (83.3%)	1 (16.7%)		
Grade III	5	3 (60%)	2 (40%)		
*ER*					
Positive	51	24 (47.1%)	27 (52.9%)	1.41	>0.05
Negative	3	3 (100%)	0 (0%)		
*Histological type*					
Endometrioid	48	22 (45.8%)	26 (54.2%)	1.31	>0.05
Nonendometrioid	8	6 (75%)	2 (25%)		
*Age*					
<55 years	16	4 (25%)	12 (75%)	6.45	<0.05^*^
≥55 years	40	25 (62.5%)	15 (37.5%)		
*Myometrial invasion*					
<1/2	43	19 (44.2%)	24 (55.8%)	1.9	>0.05
≥1/2	12	8 (66.7%)	4 (33.3%)		
*Lymph node metastasis*					
No	50	24 (48%)	26 (52%)	0.19	>0.05
Yes	6	4 (66.7%)	2 (33.3%)		

*P < 0.05.

To investigate the mechanism of NORAD downregulation in EC, bioinformatic analysis of the NORAD promoter showed that there was a CpG island (-1300 to -1475) located upstream of the transcription start site (TSS) of NORAD (Fig. 1d). The CpG island hypermethylation phenotype (CIMP) has been established as one of the hallmarks in many cancers^23,24^. Bisulfite sequencing PCR (BSP) was performed to investigate the CpG methylation status of normal, peri-tumor, and tumor tissues, including early-stage and advanced-stage tissues. Our results found that the methylation levels at the NORAD promoter were enhanced in tumor tissues compared with those in normal tissues and gradually increased with the progression of EC (Fig. 1d). To confirm these findings, we further treated ISK and SPEC-2 cells with the methyltransferase inhibitor Azacitidine (Aza). We found that Aza treatment with increasing concentration and time significantly inhibited the methylation of NORAD promoter (Fig. 1e), resulting in rescued NORAD expression in these two cell types (Fig. 1f). These results verified that the promoter hypermethylation-associated suppression of NORAD occurred in EC.

Overall, our study demonstrated that NORAD was downregulated due to its promoter hypermethylation in EC and potentially served as a biomarker for EC progression and prognosis.

### Overexpression of NORAD inhibits cell growth and promotes apoptosis in EC cells

**Fig. 2:**
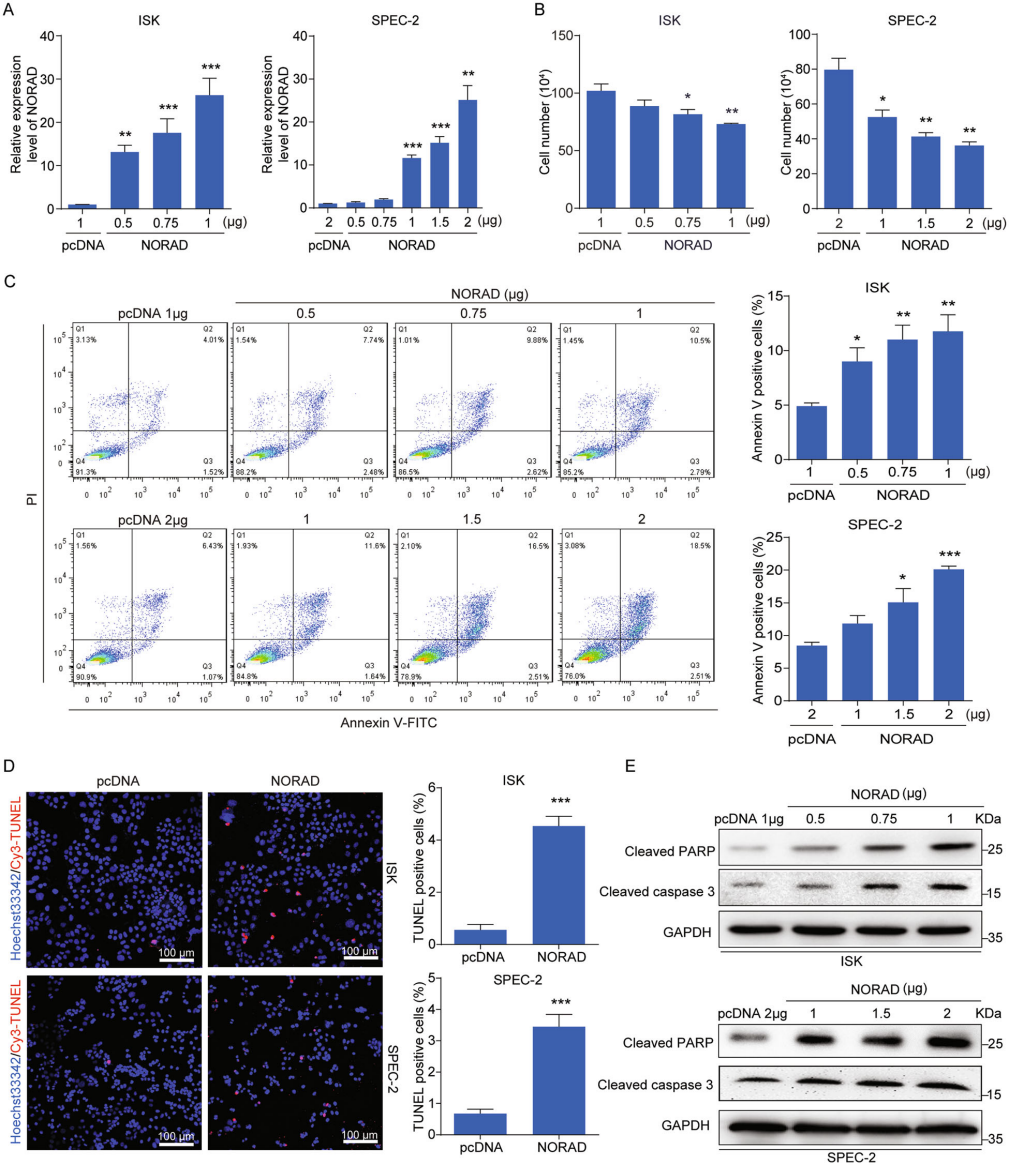
NORAD promotes apoptosis in EC cells. **a** qRT-PCR analysis for the expression level of NORAD in ISK and SPEC-2 EC cell lines with different doses transfection of NORAD, in comparison with the empty vectors. **b** Cell-counting assays for the control and ectopic NORAD-expressing ISK and SPEC-2 cells after 48 h transfection. **c** Increased percentage of apoptosis in the ectopic NORAD-expressing EC cells after 48 h transfection via FACS analysis. **d** TUNEL assays for apoptotic cells in the control and NORAD-expressing EC groups (left). Statistics of the TUNEL-Cy3 positive cells are shown (right). Scale bar, 100 μm. **e** Activated expression of cleaved PARP and cleaved caspase-3 was visualized by western blot. The results were determined from triplicates, and the error bars represented as the mean ± SD, *P < 0.05, **P < 0.01, ***P < 0.001. TUNEL TdT-mediated dUTP Nick-end labeling, GAPDH glyceraldehyde-3-phosphate dehydrogenase.

To explore the exact function of NORAD in EC, we transfected NORAD into ISK and SPEC-2 cells (Fig. 2a). Notably, we observed that overexpression of NORAD significantly inhibited the cell population (Fig. 2b). The flow-cytometry analysis (FACS) revealed that NORAD triggered EC cells apoptosis (Fig. 2c), but had no significant effect on cell-cycle progression (Supplementary Fig. S2a, b), indicating that the impairment of cell growth might primarily resulted from the induction of apoptosis by NORAD. In line with this observation, the induced apoptosis by NORAD overexpression was also judged by TUNEL assay and apoptotic markers detection (cleaved PARP and cleaved caspase-3) (Fig. 2d, e).

We further investigated the functional activity of endogenous NORAD expression rescued by Aza. Our studies first found that the cell apoptosis of EC was induced after Aza treatment for 72 h (Supplementary Fig. S3a). Then, we successfully established a serous EC patient-derived xenograft (PDX) model to study the apoptosis induction with Aza treatment in vivo, which was confirmed by hematoxylin–eosin (H&E) staining (Supplementary Fig. S3b). We found that Aza treatment did not affect the health of mice by weight supervision (Supplementary Fig. S3c), but substantially impaired tumor growth after 17 days, and eventually achieved 55.7% tumor inhibition at day 27 (Supplementary Fig. S3d). Subsequently, TUNEL and immunofluorescence staining also demonstrated that Aza treatment apparently induced cell apoptosis in EC-derived tumors (Supplementary Fig. S3e, f). Furthermore, we detected the decreased methylation level at the NORAD promoter after Aza treatment, while the NORAD expression was consequently increased treated by Aza in the PDX model (Supplementary Fig. S3g, h).

Taken together, our results demonstrated that both endogenous and exogenous NORAD expression promoted EC cell apoptosis as a tumor suppressor.

### The interaction of NORAD and FUBP1 enhances cell apoptosis in EC

**Fig. 3:**
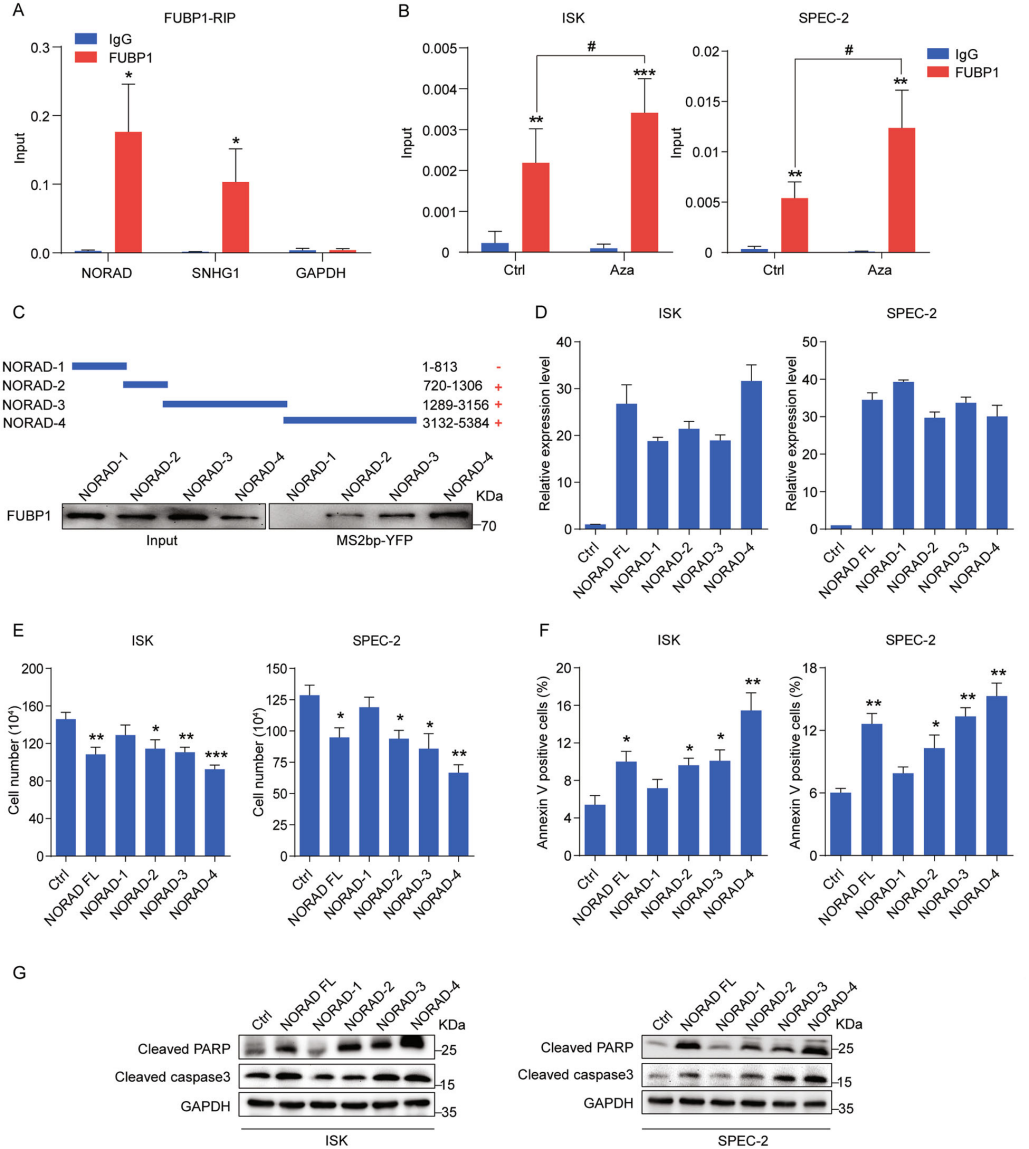
The binding of NORAD and FUBP1 is essential for NORAD to induce apoptosis. **a** qRT-PCR analysis for the binding of NORAD, SNHG1, and GAPDH expression by an anti-FUBP1 antibody compared with that by an IgG control antibody in 293FT cells. SNHG1 and GAPDH served as the positive and negative controls, respectively. **b** qRT-PCR analysis for the binding of NORAD on FUBP1 in EC cells with Aza treatment by RIP assays. **c** Western blot for the FUBP1 protein (lower panel) pulled down by truncated NORAD (NORAD-1, -2, -3, and -4; upper panel). **d** The expression levels of full-length NORAD and fragments transfected in ISK and SPEC-2 cells were detected by qRT-PCR. **e** Cell-counting assays for ISK and SPEC-2 cells transfected with full-length NORAD and fragments, respectively. **f** The percentage of apoptotic cells transfected with full-length NORAD and fragments, evaluated by FACS analysis. **g** Western blot for the expression of cleaved PARP and cleaved caspase-3 after ectopic expression of full-length NORAD and fragments. The results were determined from triplicates, and the error bars represented as the mean ± SD, */# P < 0.05, **P < 0.01, ***P < 0.001. FUBP1 far upstream element-binding protein 1, SNHG1 small nucleolar RNA host gene 1, RIP RNA immunoprecipitation, YFP yellow fluorescent protein.

To elucidate the mechanism of NORAD in apoptosis induction, we attempted to identify its cooperative proteins by exploring the mass spectrometry (MS) data^10,11^. Among various partners of NORAD, we focused on the far upstream element-binding protein 1 (FUBP1), possessing multiple binding regions on NORAD^10,11^, which is critical for antagonizing apoptosis and promoting cell survival in hepatocellular and colorectal carcinoma^20,21^. FUBP1 expression was upregulated in the EC tissues compared with normal tissues according to TCGA data (Supplementary Fig. S4a), which was consistently confirmed in the tissue sections of EC patients by immunohistochemistry (Supplementary Fig. S4b). In addition, knockdown of FUBP1 resulted in the inhibition of cell growth and induction of apoptosis (Supplementary Fig. S4c–g), indicating that FUBP1 played an important role in resistance to apoptosis in EC as well. To validate the interaction of NORAD and FUBP1, we overexpressed FUBP1 in 293FT cells and performed RNA immunoprecipitation (RIP) to detect FUBP1 binding with NORAD and SNHG1 (FUBP1-binding RNA)^25^ (Fig. 3a). Moreover, we performed RIP assays in EC cells under the condition of endogenous NORAD expression rescued by Aza, which showed the increased binding of NORAD on FUBP1 (Fig. 3b). To further clarify the specific regions of NORAD responsible for FUBP1 binding, we divided NORAD into four fragments (NORAD-1, 2, 3, and 4) according to the predicted peaks of the FUBP1-binding regions in the MS data^10^. These four fragments of NORAD were tagged with an MS2 sequence and co-transfected with FUBP1 and MS2bp-YFP (fused by MS2-binding protein and yellow fluorescent protein) into 293FT cells to perform RNA pull-down assays (Fig. 3c). Our results showed that three fragments of NORAD (NORAD-2, 3, and 4), rather than NORAD-1, could bind to FUBP1, suggesting that there were multiple FUBP1-binding sites distributed on NORAD. To elicit the significance of the NORAD/FUBP1 interaction, we transfected the full-length and four fragments of NORAD into EC cells (Fig. 3d) and found that the overexpression of NORAD full-length, NORAD-2, NORAD-3, and NORAD-4, which bound to FUBP1, significantly inhibited the number of EC cells (Fig. 3e) and enhanced cell apoptosis (Fig. 3f, g). However, the NORAD-1 fragment with no interaction of FUBP1 had no impact on the cell growth and apoptosis of EC cells (Fig. 3e–g). These results implied that the interaction with FUBP1 was responsible for NORAD to promote cell apoptosis in EC.

### NORAD-4 rescues apoptosis inhibition and tumor growth mediated by knockdown of NORAD in vitro and in vivo

**Fig. 4:**
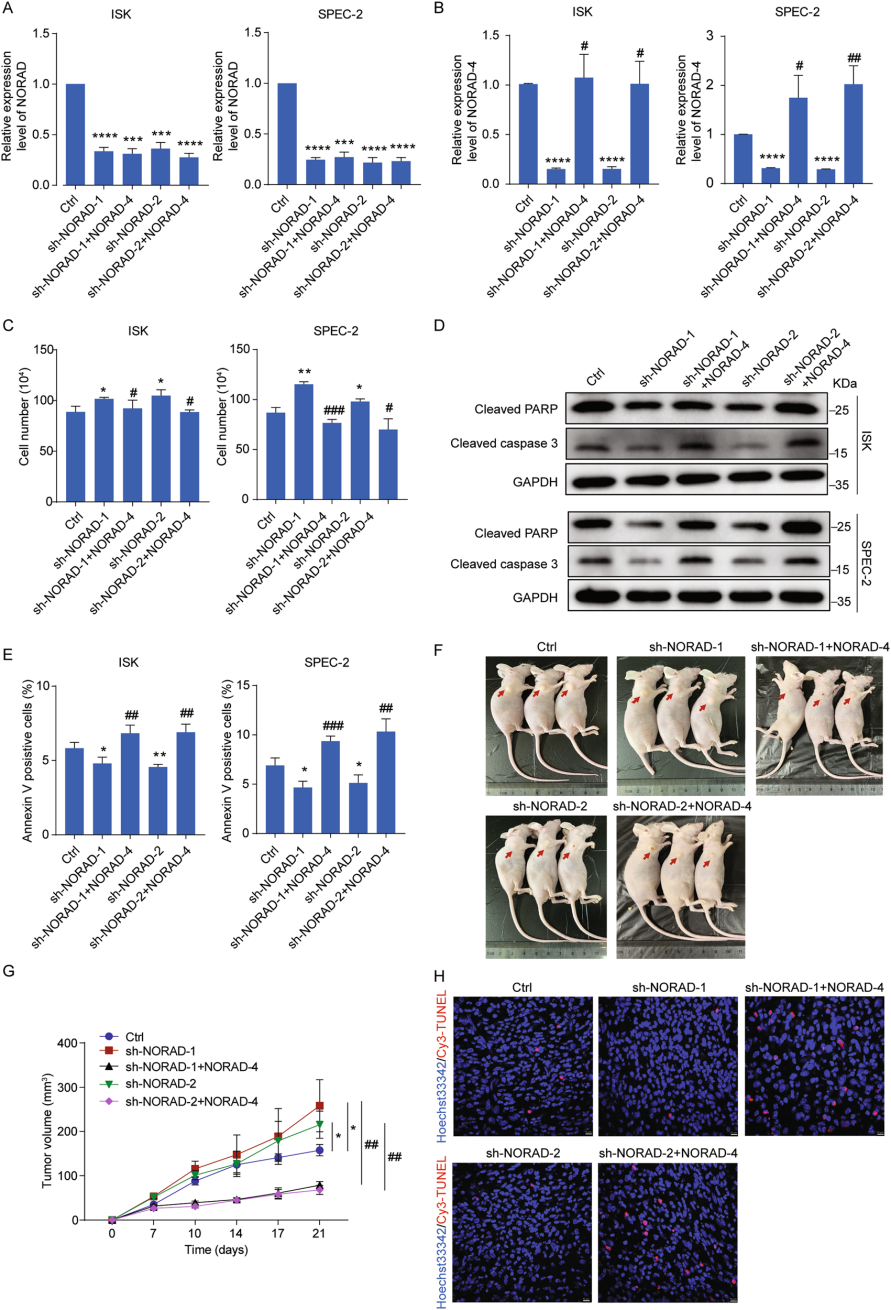
NORAD-4 rescues the apoptosis inhibition and tumor growth mediated by knockdown of NORAD in vitro and in vivo. **a**, **b** The expression level of NORAD (**a**) and NORAD-4 (**b**) was detected by qRT-PCR in NORAD knockdown cell lines and in NORAD knockdown rescued by NORAD-4 fragment cell lines. **c** Cell-counting assays for the NORAD knockdown and rescued by NORAD-4 cell lines. **d** The expression levels of apoptotic associated markers were detected by western blot in the NORAD knockdown and rescued by NORAD-4 cell lines. **e** The percentage of apoptotic cells in the NORAD knockdown and rescued by NORAD-4 cell lines was analyzed by FACS. **f** SPEC-2-derived cell lines (sh-NORAD-1/2, sh-NORAD-1/2 + NORAD-4) were subcutaneously injected into the hind flanks of nude mice. **g** Tumor volume was monitored from day 0 to day 21 post injection. **h** Apoptosis in tumor tissues was presented by TUNEL assay. Scale bar, 10 μm. The results were determined from triplicates, and the error bars represented as the mean ± SD, *^/#^*P* < 0.05, *^/##^*P* < 0.01, ***^/###^*P* < 0.001, ****P < 0.0001.

To further understand the key role of NORAD in EC progression, we first constructed the NORAD knockdown cell lines rescued by NORAD-4 fragment (bound with FUBP1) (Fig. 4a, b). Knockdown of NORAD promoted cell growth (Fig. 4c) and inhibited cell apoptosis performed by FACS and detection of apoptotic proteins in EC cells (Fig. 4d, e). However, introduction of NORAD-4 fragment successfully reversed cell growth and apoptosis inhibition in NORAD knockdown cells (Fig. 4c–e). In addition, we introduced the NORAD-4 rescued cell lines into the xenograft mice model. Knockdown of NORAD resulted in excessive tumor growth and reduced apoptosis (Fig. 4f–h). While introduction of NORAD-4 fragment significantly impaired the tumor growth and cell apoptosis inhibition mediated by knockdown of NORAD in vivo (Fig. 4f–h). In conclusion, NORAD played a key role in EC progression via interacting with FUBP1.

### NORAD affects the cytosol–nuclear trafficking of FUBP1 through its central domain

**Fig. 5:**
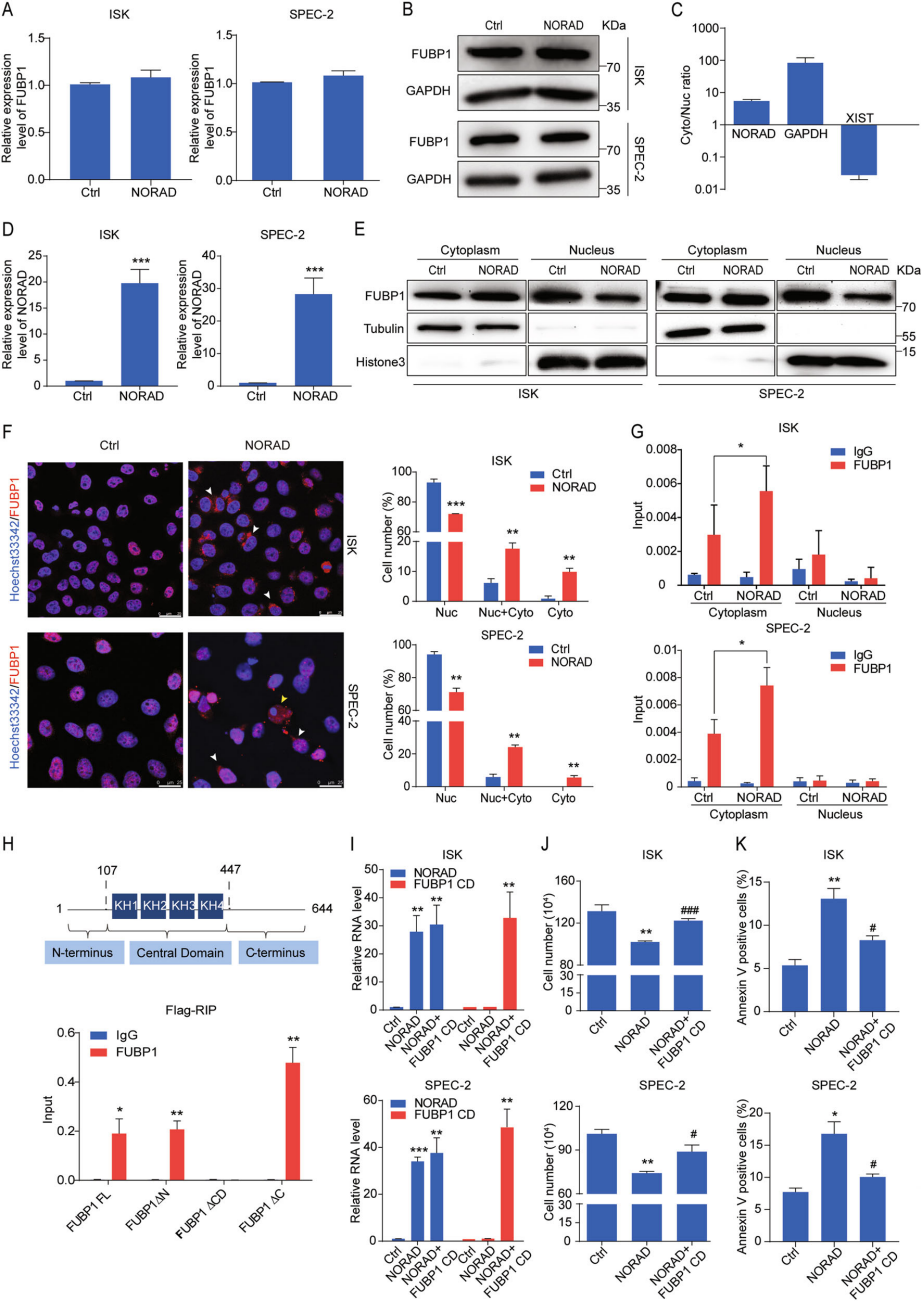
NORAD impairs the nuclear localization of FUBP1 through its central domain. **a**, **b** The FUBP1 expression level after the introduction of NORAD in ISK and SPEC-2 cells via qRT-PCR (**a**) and western blot (**b**). **c** The subcellular distribution of NORAD was analyzed by qRT-PCR. GAPDH and XIST genes were used as controls for the cytoplasmic and nuclear fractions, respectively. **d** The expression level of NORAD was detected by qRT-PCR. **e** The fractionation of FUBP1 was visualized by western blot after ectopic expression of NORAD in ISK and SPEC-2 cells. GAPDH and Histone 3 indicated the cytoplasmic and nuclear fractions, respectively. **f** Immunofluorescence assays indicated the altered localization of FUBP1 (red) after introduction of NORAD in ISK and SPEC-2 cells (left). Quantifications of the percentages of FUBP1 presented only in the nucleus, in the cytoplasm, and both in the nucleus and cytoplasm are shown (right). White arrows represented the cells which FUBP1 was distributed both in the cytoplasm and nucleus. Yellow arrows represented the cells in which FUBP1 was distributed only in the cytoplasm. Scale bar, 25 μm. **g** The subcellular fractionation followed by RIP assays was performed to analyze the interaction of NORAD and FUBP1 in the cytoplasmic and nuclear lysates of NORAD overexpressing cells. **h** qRT-PCR analysis of NORAD immunoprecipitated by Flag-tagged full-length and three deleted mutations of FUBP1 in 293FT cells compared with the IgG control. **i** The expression level of NORAD and FUBP1 CD truncation was detected by qRT-PCR. **j** Cell-counting assays of the rescued cell growth by FUBP1 CD in the NORAD-expressing ISK and SPEC-2 cells. **k** FACS analysis of the rescued percentage of apoptotic cells by FUBP1 CD in the NORAD-expressing ISK and SPEC-2 cells. The results were determined from triplicates, and the error bars represented as the mean ± SD, *^/#^*P* < 0.05, **P < 0.01, ***^/###^*P* < 0.001. XIST X inactivation-specific transcript.

We next focused on investigating how the NORAD/FUBP1 interaction induced apoptosis. We first noticed that overexpression of NORAD was unable to modulate the mRNA or protein level of FUBP1 (Fig. 5a, b). FUBP1 is often recognized as a nuclear protein based on its recognition ability of FUSE element^17^, while increasing evidences indicate that FUBP1 can interact with cytoplasmic RNAs^26,27^. Cell fractionation followed by RT-qPCR revealed that NORAD was predominately located in the cytoplasm (Fig. 5c), suggesting that the interaction of NORAD and FUBP1 might occur in the cytoplasm. Notably, we identified that overexpression of NORAD attenuated FUBP1 nuclear accumulation by subcellular fractionation followed by western blot (Fig. 5d, e). We subsequently performed immunofluorescence staining to analyze the ratio of FUBP1 distribution only in the cytoplasm, nucleus, or both (Fig. 5f). The statistics indicated that overexpression of NORAD apparently impaired the nuclear localization of FUBP1, suggesting that the convergence of NORAD and FUBP1 altered FUBP1 subcellular localization. Consequently, we performed the subcellular fractionation followed by RIP assays. Our results identified that overexpression of NORAD significantly enhanced the interaction with FUBP1 in the cytoplasm, resulting in the impairment of FUBP1 localized in nucleus (Fig. 5g). Next, to elucidate the specific region of FUBP1 binding with NORAD, we constructed three depletion mutants of FUBP1 (FUBP1 ΔN, ΔCD, and ΔC) according to its functional domains (including the N-terminal inhibitory domain, central domain, and C-terminal transactivation domain)^17,18^. Our results showed that only the deletion of FUBP1 central domain abolished its interaction with NORAD (Fig. 5h). The central domain of FUBP1 possesses a dual role in DNA and RNA binding^17,18^. Accordingly, we ascertained that NORAD prevented the nuclear translocation of FUBP1 by binding with its central domain as a “decoy”. Then, we co-transfected NORAD and the central domain of FUBP1 fragment (FUBP1 CD) into ISK and SPEC-2 cells (Fig. 5i). We found that overexpression of the FUBP1 CD fragment significantly reversed the cell growth inhibition and apoptosis induction mediated by NORAD (Fig. 5j, k), indicating that the dominant-negative fragment of FUBP1 CD competitively bound with NORAD in the cytosol to facilitate endogenous FUBP1 translocation into the nucleus, where it rescued cell viability.

### NORAD/FUBP1 interaction regulates the downstream pro-apoptotic genes

**Fig. 6:**
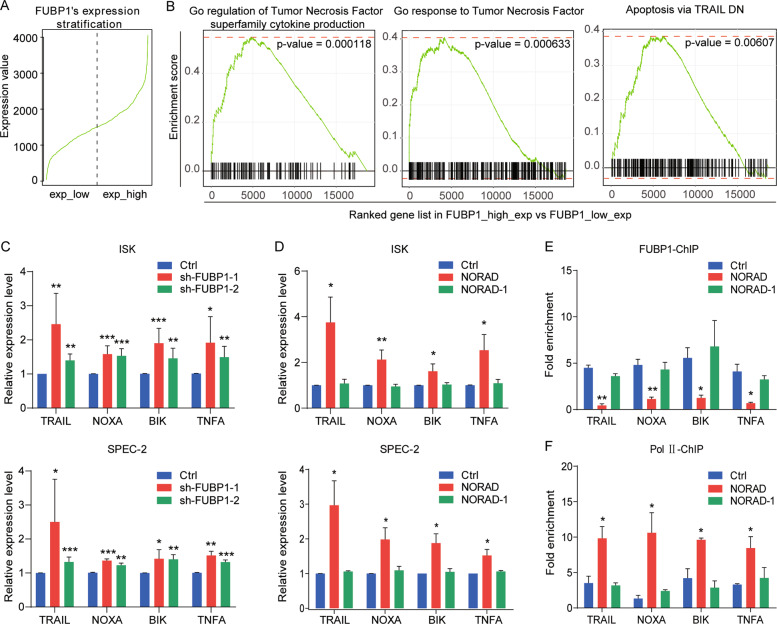
The NORAD/FUBP1 interaction results in the upregulation of downstream pro-apoptotic genes. **a** RNA-seq data of liver cancer from TCGA to classify FUBP1 high- and low-expressed groups. **b** GSEA for the FUBP1-related pathways in liver cancer. **c** qRT-PCR analysis for the expression of four FUBP1 downstream targets (TRAIL, NOXA, BIK, and TNFA) by knockdown of FUBP1. **d** qRT-PCR analysis for the expression of four FUBP1 downstream target genes by the introduction of full-length NORAD or the NORAD-1 fragment, which was not bound to FUBP1. **e**, **f** ChIP-qPCR analysis for the FUBP1 (**e**) and RNA polymerase II (**f**) occupancies at the promoters of four target genes (TRAIL, NOXA, BIK, and TNFA) after transfecting full-length NORAD or NORAD-1 fragment. The fold enrichment was relative to the input DNA. The results were determined from triplicates, and the error bars represented as the mean ± SD, *P < 0.05, **P < 0.01, ***P < 0.001. TNF tumor necrosis factor, TRAIL TNF-related apoptosis-inducing ligand, NOXA PMAIP1, phorbol-12-myristate-13-acetate-induced protein 1, BIK BCL2 interacting killer, ChIP chromatin immunoprecipitation, Pol II RNA polymerase II.

We further explored the downstream targets of FUBP1 to execute its anti-apoptotic effect. Gene set enrichment analysis (GSEA) showed that upon the high and low expression of FUBP1 in liver cancer, downstream genes were evidently relevant to the apoptosis pathway (Fig. 6a, b). Previous studies have reported that FUBP1 suppressed the transcription of the pro-apoptotic genes (such as BIK, NOXA, TRAIL, and TNFA)^21^. Our findings showed that knockdown of FUBP1 indeed upregulated the expression level of these four genes (Fig. 6c), which were also promoted by NORAD overexpression (Fig. 6d), indicating that these pro-apoptotic genes were co-regulated by NORAD and FUBP1. Chromatin Immunoprecipitation (ChIP) assay identified that overexpression of NORAD significantly reduced the FUBP1 occupancies on these four gene promoters (Fig. 6e). Consistently, the enrichment of RNA polymerase II on these gene promoters was enhanced after NORAD overexpression (Fig. 6f). Moreover, we transfected NORAD-1 (without interaction with FUBP1), which had no impact on the expression, FUBP1 occupancy, or transcription activity of these downstream target genes (Fig. 6d–f). Taken together, we demonstrated that the interaction of NORAD and FUBP1 affected the nuclear distribution of FUBP1 and facilitated its downstream pro-apoptotic gene transcription, eventually resulting in apoptosis induction in EC cells.

## Discussion

LncRNAs have been recently emerged as important players in modulating tumor initiation, progression, and the assessment of prognosis^4–6^. NORAD was recently discovered to promote cancer cells proliferation, invasion, and metastasis in various cancers (such as bladder, pancreatic, cervical cancer, etc.)^13,14,28,29^. While, NORAD retained controversial roles in liver, lung, and breast cancer^16,30^, implying that NORAD acted on tumorigenesis and progression in a context-dependent manner. In our study, we analyzed the public data in TCGA and collected the tumor tissues of EC patients, which illustrated that NORAD was downregulated due to promoter hypermethylation in EC patients compared with normal tissues. Moreover, epigenetic inactivation of NORAD was relevant to adverse progression and poor prognosis. Therefore, for the first time, we propose NORAD as a potential molecular marker for the clinical assessment of EC progression and prognosis.

Normal cells suffering from external or internal stress will trigger DNA damage and repair^31^. Once defective cells failed to repair effectively, cell death pathways (apoptosis, necrosis, or autophagy) would be activated to eliminate the negative effects of genomic toxicity on cells^32,33^. However, during the process of tumor initiation, these defective cells could bypass the cell death pathway to complete the malignant transition, which might be the important cause of tumor formation^31^. Previous studies revealed that NORAD bound with PUMILIO1/2 or RBMX to maintain genomic stability^9–11^. However, inactivation of NORAD downregulated the expression of genes associated with DNA damage and repair, such as RBMX and PARP1, resulting in genomic instability^10^. Our study indicated that the expression of NORAD was decreased in the transition from normal endometrial tissue to EC tissue. Knockdown of NORAD could promote tumor growth and prevent cell apoptosis in vitro and in vivo. While, both exogenous NORAD overexpression and rescued endogenous NORAD expression by Aza could inhibit cell growth and promote cell apoptosis, as a tumor suppressor. Thus, these results indicated that NORAD was critically involved in the balance between cell proliferation and apoptosis evasion in EC progression.

Cytoplasmic distributed lncRNAs can execute their regulatory roles through binding with proteins to affect their function, subcellular localization, or protein–protein interaction^4,34,35^. NORAD contains multiple repetitive motifs and serves as a molecular decoy for the PUMILIO protein, indicating that NORAD might function as a platform for assembling proteins^10^. In view of FUBP1 function in EC and its multiple binding regions on NORAD in the MS data^10,11^, we confirmed the interaction of NORAD and FUBP1 under exogenous and endogenous conditions in EC, and identified FUBP1 binding with at least three regions of NORAD. We also revealed that binding with FUBP1 was essential for NORAD to induce apoptosis in EC cells. Furthermore, introduction of NORAD-4 fragment (bound with FUBP1) could reverse cell growth and apoptosis inhibition mediated by knockdown of NORAD in vitro and in vivo. These findings suggested that NORAD had the capacity to interact with multiple FUBP1 proteins as a decoy to regulate cell apoptosis.

To date, FUBP1 was ascertained to be upregulated in colorectal and hepatocellular cancer^20,21^. Our study also revealed that FUBP1 was upregulated in EC, and knockdown of FUBP1 remarkably enhanced cell apoptosis. TAL1 was reported to bind to the FUBP1 promoter and activate its transcription in erythroid differentiation^36^. FUBP1 was also regulated by the PI3K/AKT/mTOR pathway and caspase protein in liver cancer^37^ or targeted by miR-16 in breast and gastric cancer^38^. In addition, FUBP1 was found to be ubiquitinated by p38 in lung cell differentiation or identified as a substrate of parkin in Parkinson’s disease^39,40^. Intriguingly, our results showed that NORAD had no impact on the transcription or stability of FUBP1 but attenuated FUBP1 nuclear enrichment, which impaired its occupancies on the promoters of downstream pro-apoptotic genes. Our results further found that FUBP1 interacted with NORAD through its central domain (DNA-/RNA-binding region). Overexpression of the FUBP1 central domain could competitively bind with NORAD and facilitate endogenous FUBP1 trafficking into the nucleus to reverse the cell apoptosis induction mediated by NORAD. FUBP1, primarily located in the nucleus, was prevented to be imported into the nucleus due to caspase-3/7 cleavage during the breast and cervical cancer cells apoptosis^41^. Herein, we provided an optional mechanism that NORAD decoyed FUBP1 in the cytosol and impaired its translocation to the nucleus, which was responsible for apoptosis induction in EC.

In conclusion, we elucidate that epigenetic inactivation of NORAD affects the cytosol–nucleus trafficking of the anti-apoptotic factor FUBP1 and the expression of its target pro-apoptotic genes, resulting in EC cells evasion from apoptosis. In this regard, investigating NORAD crosstalk will lead us to significant insights into the mechanism of EC progression. Moreover, we are the first to highlight the predictive clinical value of NORAD as an EC diagnostic and prognostic biomarker and the possibility of developing NORAD-targeted therapy.

## Materials and methods

### Sample collections from patients

EC (*n* = 56) tissues were collected from patients who underwent hysterectomy at the Tongji University Affiliated Shanghai First Maternity and Infant Hospital (Shanghai, China) from 2015 to 2019. Peri-tumor endometrial tissues (*n* = 54) were sampled 1–2 cm away from tumors in the surgeries^42^. Normal endometrial specimen (*n* = 20) were collected from women undergoing non-maligmant diseases (uterine leiomyoma) with no underlying endometrial pathology^43^. The histology of all tissues was verified by two independent pathologists. No patients had undergone endocrine therapy, radiotherapy, or chemotherapy before surgery. All patients have signed the informed consent form before collection. The detailed clinical information of these patients was provided in Supplementary Table S1. The research project was approved by the Human Investigation Ethical Committee of Tongji University Affiliated Shanghai First Maternity and Infant Hospital.

### RNA immunoprecipitation (RIP)

RIP was performed as previously described^44^. A total of 5 × 10^6^ cells were lysed with lysate buffer. Protein A Magnetic Beads (161-4013, Bio-Rad) and Protein G Magnetic Beads (161-4023, Bio-Rad) were incubated with 3 μg of antibodies, rotating for at least 6 h. Lysates were added to the prepared beads in RIP buffer, rotating overnight for immunoprecipitation. Finally, RNA was extracted with RNAiso Plus Reagent. The antibodies in RIP assay were followed as: anti-FUBP1 (ab192867, Abcam), anti-IgG-Rb (#2729, Cell Signaling Technology), and anti-flag-Rb (14793s, Cell Signaling Technology).

### Xenograft mice model

Five-week-old female BALB/c nude mice were purchased from the National Resource Center for Rodent Laboratory Animals of China. The mice used in animal studies were randomly and blindly allocated into experimental and control group. Five SPEC-2-derived cell lines (Ctrl, sh-NORAD-1, sh-NORAD-2, sh-NORAD-1 + NORAD-4, sh-NORAD-2 + NORAD-4), suspended at the concentration of 1 × 10^7^ cells in 100 μL of PBS, were subcutaneously injected into the hind flanks of nude mice (*n* = 3, each group). On the 7th day after injection, mice were monitored and the tumor volume was calculated using the formula 1/2 (length × width^2^) twice a week. The mice were sacrificed at day 21 post injection. These studies were approved by the Institutional Animal Care and Use Committee of Tongji University (no. TJLAC-019-103).

### Statistical analysis

The statistical analyses were performed with GraphPad Prism 7 software. The results were from triplicate experiments, and the data was presented as the mean ± SEM or mean ± SD. The significance of mean values was determined by unpaired two-tailed Student’s *t* test. Pearson’s chi-square test and nonparametric test were used to analyze the clinical variables. The survival times of different groups of patients were analyzed using the Kaplan-method with the log-rank test. *^/#^, **^/##^, ***^/###^, and **** represent *P* < 0.05, *P* < 0.01, *P* < 0.001, and *P* < 0.0001, respectively.

## Supplementary information

Supplementary Information

Supplementary Information

Supplementary Information

Supplementary Information

Supplementary Information

Supplementary Information

Supplementary Information
