# Supplementary figures and images for: NORAD orchestrates endometrial cancer progression by sequestering FUBP1 nuclear localization to promote cell apoptosis
Source: Cell Death Dis. 2020 Jun 18;11(6):473. doi: 10.1038/s41419-020-2674-y (PMC7303217; doi:10.1038/s41419-020-2674-y)

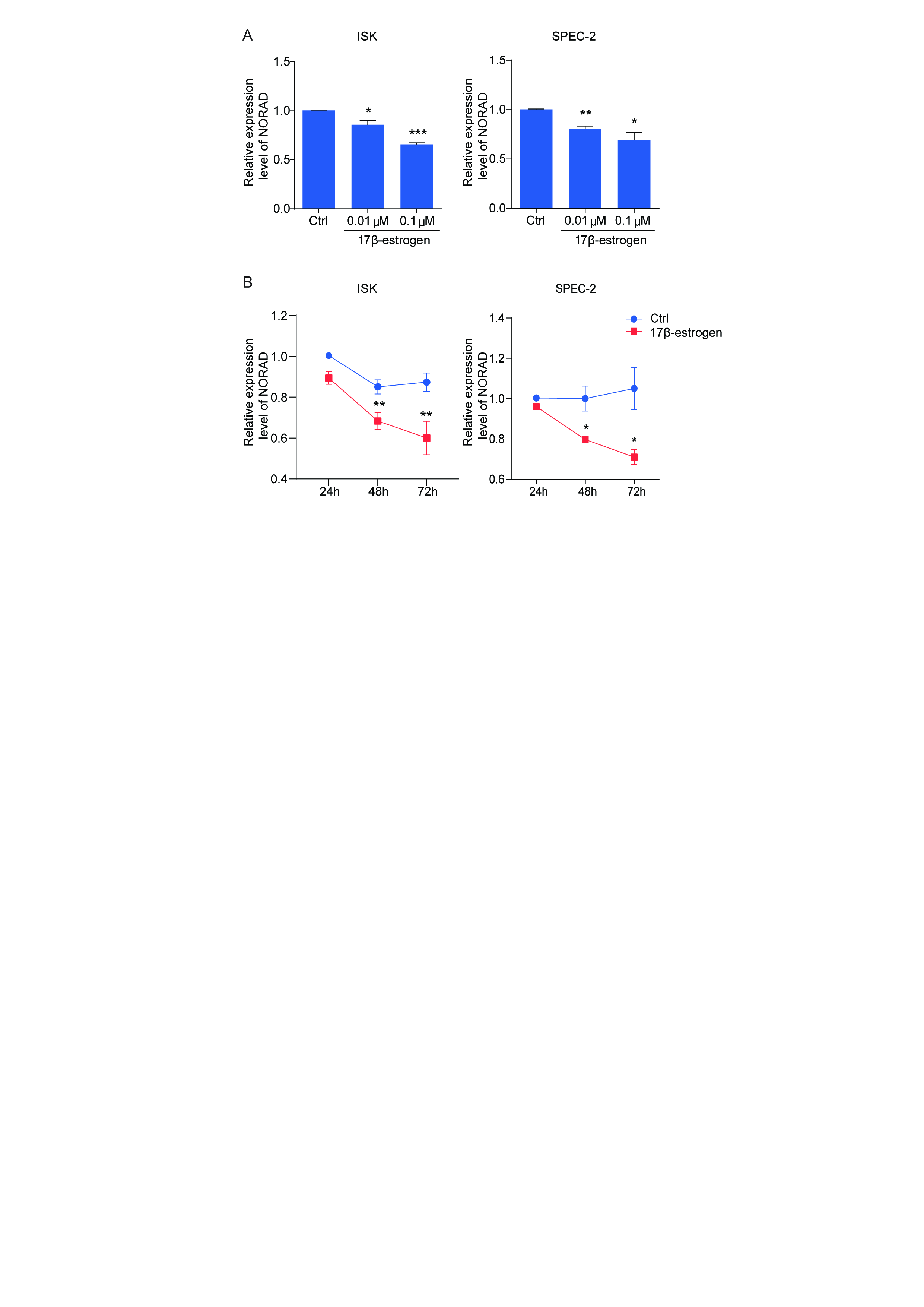

Supplement: Supplementary file 1 — Supplementary Information [file 41419_2020_2674_MOESM1_ESM.tif]

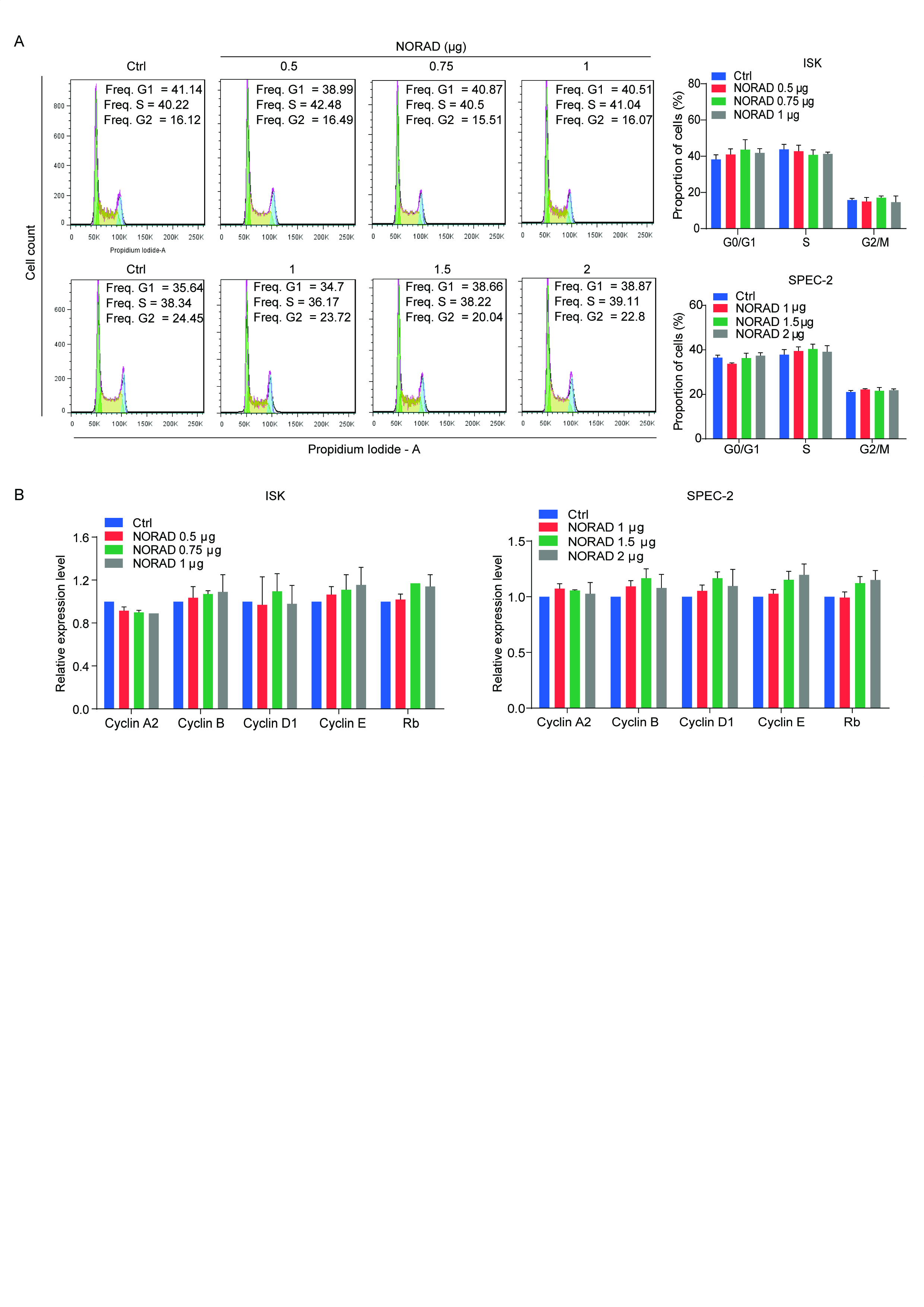

Supplement: Supplementary file 2 — Supplementary Information [file 41419_2020_2674_MOESM2_ESM.tif]

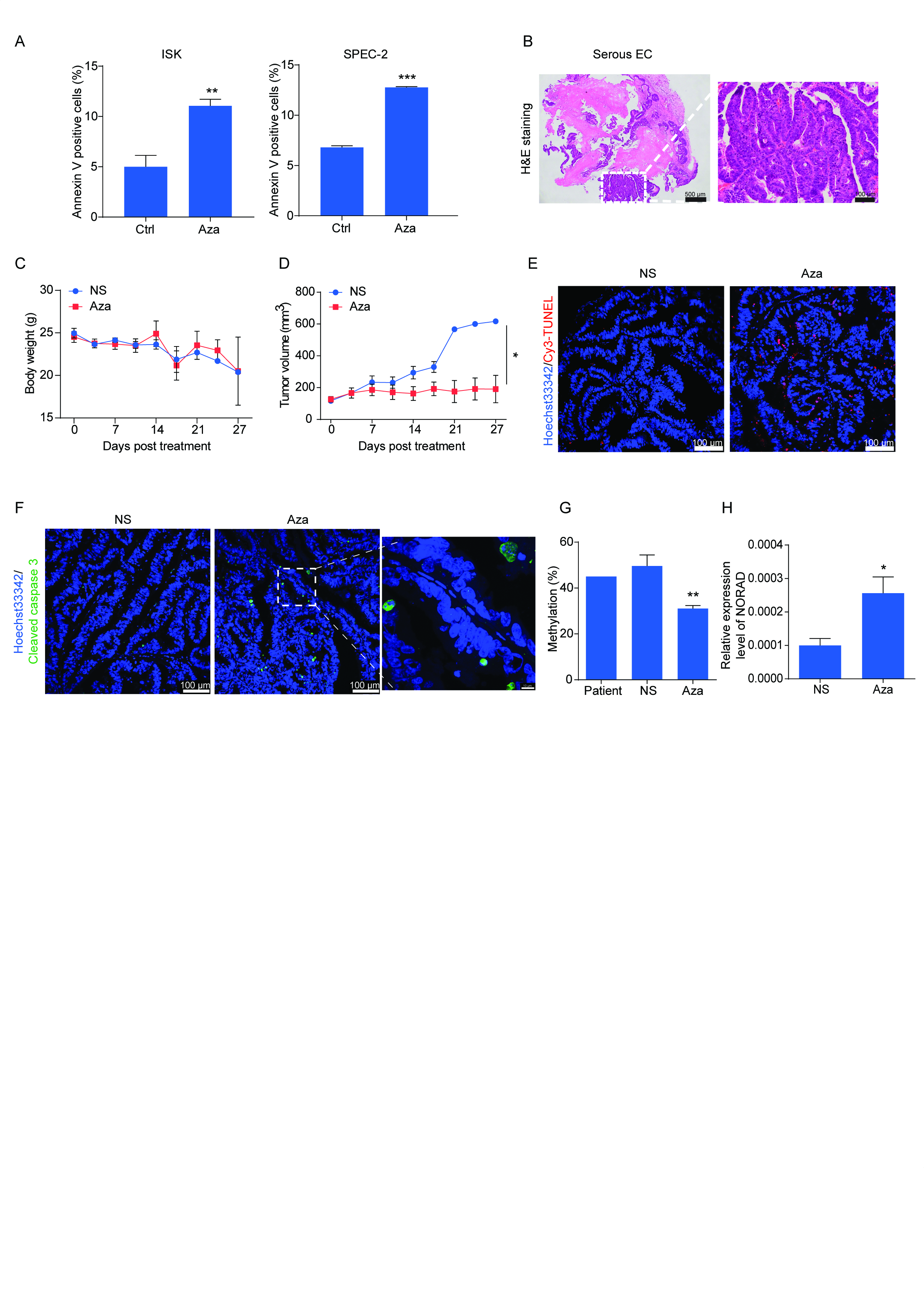

Supplement: Supplementary file 3 — Supplementary Information [file 41419_2020_2674_MOESM3_ESM.tif]

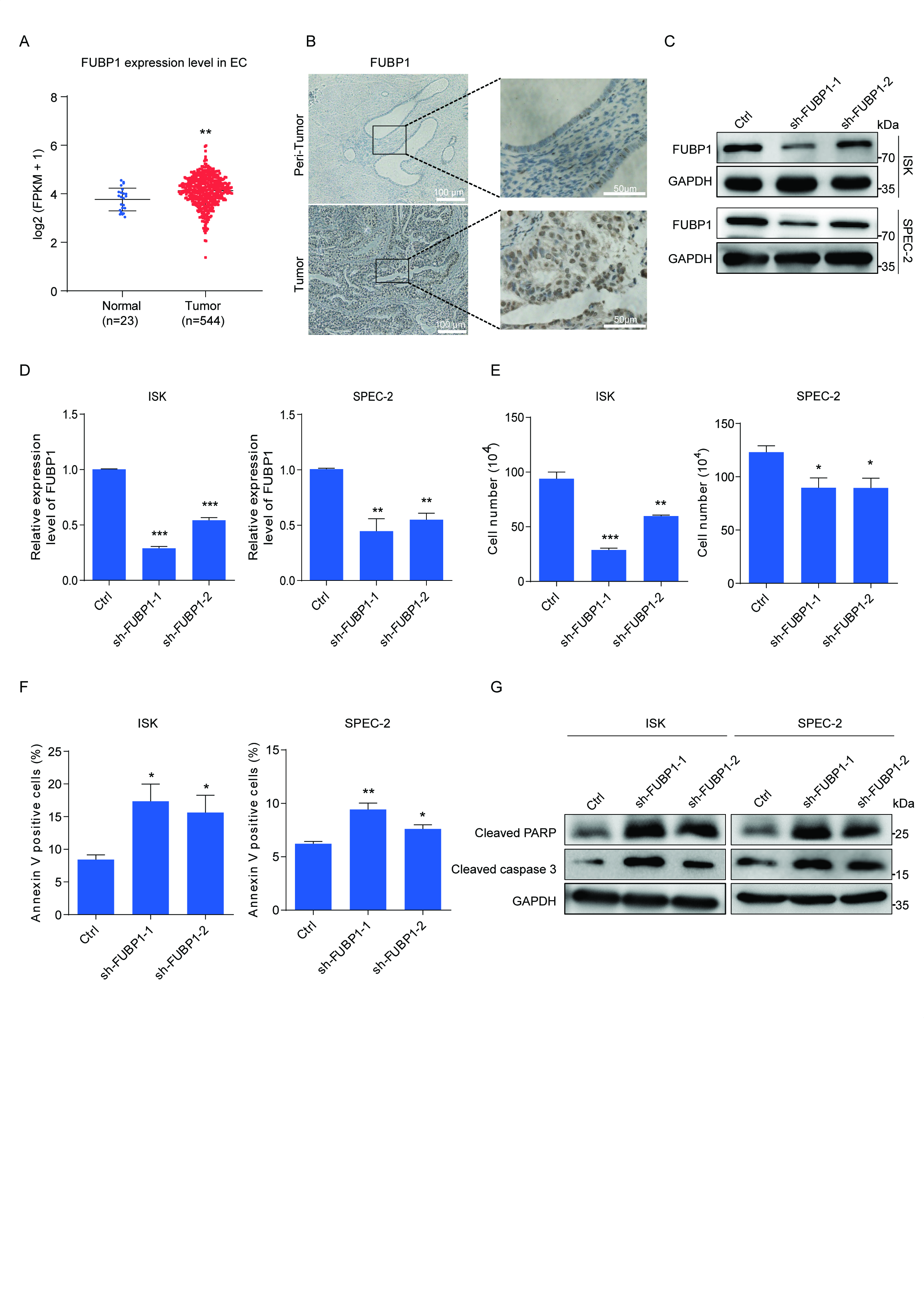

Supplement: Supplementary file 4 — Supplementary Information [file 41419_2020_2674_MOESM4_ESM.tif]
