# Supplementary material for: NORAD orchestrates endometrial cancer progression by sequestering FUBP1 nuclear localization to promote cell apoptosis
Source: Cell Death Dis. 2020 Jun 18;11(6):473. doi: 10.1038/s41419-020-2674-y (PMC7303217; doi:10.1038/s41419-020-2674-y)
Supplement: Supplementary file 5 — Supplementary Information [file 41419_2020_2674_MOESM5_ESM.docx]

**Supplementary Data**

**Supplementary Figure Legends**

Supplementary Figure S1. Estrogen-dependent downregulation of NORAD.

a,b. qRT-PCR analysis of NORAD expression after treatment with 17β-estrogen at different doses and times in ISK and SPEC-2 cells.

The results were determined from triplicates and the error bars represented as the Mean ± SD, * *P* < 0.05, ** *P* < 0.01, *** *P* < 0.001.

Supplementary Figure S2. NORAD has no effect on the cell-cycle distributions of EC cells.

1. Quantification of the cell-cycle distributions after ectopic NORAD expression in ISK and SPEC-2 cells by FACS.
2. qRT-PCR analysis of the cell cycle-related genes (cyclins and Rb) in control and NORAD-expressing cells.

The results were determined from triplicates and the error bars represented as the Mean ± SD.

Supplementary Figure S3. The application of Azacitidine (Aza) in inhibiting tumor growth and triggering apoptosis in EC PDX model.

a. FACS analysis of cell apoptosis in ISK and SPEC-2 cells with Aza treatment for 72 h.

b. The successfully established serous EC PDX model was confirmed by pathological histology and H&E staining. Scale bar, 500 μm or 100 μm (enlarged).

c,d. The body weight of PDX mice (c) and tumor volume of tumor xenografts (d) were supervised after treatment with Aza in comparison with those of the control group (NS).

e. TUNEL assays for apoptotic cells (Cy3-positive; red) in the tumor xenograft group after treatment with Aza in comparison with that in the NS group. Scale bar, 100 μm.

f. Representative images for immunofluorescence staining of cleaved caspase 3 (green) in tumor xenografts after treatment with Aza. Scale bar, 100 μm or 10 μm (enlarged).

g. The methylation status of the NORAD promoter in PDX tumors after treatment with Aza was determined by bisulfite sequencing in comparison with tumor tissues derived from the patient and NS group.

h. qRT-PCR analysis of NORAD expression in the tumor xenograft group after treatment with Aza in comparison with that in the NS group. Internal control, 18S.

The results were determined from triplicates and the error bars represented as the Mean ± SD, * *P* < 0.05, ** *P* < 0.01, *** *P* < 0.001. NS: Normal Saline.

Supplementary Figure S4. FUBP1 serves as an anti-apoptosis factor in EC.

1. Relative FUBP1 expression in the TCGA EC patient cohort compared with that in normal tissues.
2. Representative images for immunohistochemistry of FUBP1 expression in tumor tissues compared with that in peri-tumors from EC patients. Scale bar, 100 μm or 50 μm (enlarged).

c,d. Western blot (c) and qRT-PCR (d) confirmed the knockdown of FUBP1.

e. Cell counting assays for FUBP1 knockdown in ISK and SPEC-2 cells.

f. FACS analysis for cell apoptosis after FUBP1 knockdown in ISK and SPEC-2 cells.

g. Western blot for the expression of cleaved PARP and cleaved caspase 3 after FUBP1 knockdown in ISK and SPEC-2 cells.

The results were determined from triplicates and the error bars represented as the Mean ± SD, * *P* < 0.05, ** *P* < 0.01, *** *P* < 0.001.

**Supplementary Table Legends**

Supplementary Table S1. Clinical characteristics of EC patients.

Supplementary Table S2. Sequences of primers used for qRT-PCR in this study.

Supplementary Table S3. Sequences of primers used for ChIP-qPCR in this study.

Supplementary Table S4. Sequences of primers used for plasmid construction in this study.

Supplementary Table S5. Sequences of primers used for bisulfite sequencing in this study.
