# Supplementary material for: NORAD orchestrates endometrial cancer progression by sequestering FUBP1 nuclear localization to promote cell apoptosis
Source: Cell Death Dis. 2020 Jun 18;11(6):473. doi: 10.1038/s41419-020-2674-y (PMC7303217; doi:10.1038/s41419-020-2674-y)
Supplement: Supplementary file 6 — Supplementary Information [file 41419_2020_2674_MOESM6_ESM.docx]

**Supplementary materials and methods.**

**Cell culture and chemical agents**

The human EC cell lines Ishikawa (ISK), SPEC-2, melanoma cell line A375, colorectal cancer cell line HCT116, and 293FT were purchased from the American Type Culture Collection (ATCC; Manassas, VA, USA). The ISK cell line was cultured in Dulbecco’s modified Eagle’s medium (DMEM)/F12 (Gibco, Auckland, New Zealand). SPEC-2 was cultured in Eagle’s minimum essential medium (MEM) supplemented with 100 mM sodium pyruvate and 1.2 g/L NaHCO_3_. 293FT, A375, and HCT116 cells were cultured in DMEM (Gibco, Auckland, New Zealand). All cell culture media were supplemented with 10% fetal bovine serum (FBS) (Gibco Life Technologies, Carlsbad, CA, USA), and the cells were maintained at 37°C with 5% CO_2_ in a humidified incubator and treated with the following chemical agents: 17β-estrogen (E2758, Sigma-Aldrich) and Azacitidine (S1782, Selleck). The source of cell lines were recently authenticated by STR profiling and tested for mycoplasma contamination.

**Plasmid construction**

The full length of NORAD (NR_027451) cloned into the pcDNA3.1 vector was a gift from Joshua T. Mendell’s laboratory. The cDNA fragments corresponding to the NORAD-1, NORAD-2, NORAD-3, and NORAD-4 transcripts were cloned into the pMS2 vector for the MS2bp-mediated RNA pull-down assay^44^. The full length of Flag-tagged FUBP1, its three deletion mutants (FUBP1 ΔN, ΔCD, and ΔC) (constructed by Hanyinbt, Shanghai, China) and FUBP1 CD fragment were cloned into the pcDNA3.1 vector (Addgene). The short hairpin RNAs (shRNAs) targeted to NORAD and FUBP1 were inserted into the pLKO.1 vector (Addgene). All constructed plasmids were verified by DNA sequencing. The PCR primer sequences used for plasmid construction are shown in Supplementary Table S4.

**Stable cell line establishment**

Cells were transfected with plasmids in Opti-MEM (Gibco) using FuGENE HD (Roche). For stable knockdown of NORAD and FUBP1, shRNA-viral supernatant collected after 48 h transfection was used to infect ISK and SPEC-2 cells in 6-well plates in the presence of polybrene (8 μg/mL). ISK and SPEC-2 were selected with 1 μg/mL puromycin (Invitrogen, Carlsbad CA, USA).

**Cell growth assays, cell cycle, and apoptosis analyses**

Cells (2.5×10^5^ per well) were seeded into a 6-well plate, and the plasmids were transfected into cells after 24 h. The cell numbers were measured by Cell Counter Star after 48 h. Harvested cells were stained using an Annexin V/propidium iodide (PI) kit (KGA107, Keygen Biotech, Jiangsu, China) for apoptosis analysis. The harvested cells fixed in cold 70% ethanol overnight were stained with a PI/RNase kit (Beyotime, Shanghai, China). Samples were subjected to flow cytometry using a FACS Calibur (Becton Dickinson Biosciences, Franklin Lakes, NJ, USA) and analyzed by FlowJo software. The cells and sections of tumor tissues were subjected to a one-step TUNEL Apoptosis Assay Kit (C1090, Beyotime, Shanghai, China).

**MS2bp-YFP RNA pull-down assay**

The MS2bp-based pull-down assay was performed as described previously^44^. Cells were co-transfected with pMS2-NORAD, MS2bp-YFP, and FUBP1 vectors. After 48 h, the cells were crosslinked with 37% formaldehyde for 10 min at room temperature, followed by 1.25 M glycine quenching for 5 min. Then, cells were lysed with lysate buffer for 30 min on ice. The proteins were immunoprecipitated with a control IgG-Rb (#2729, Cell Signaling Technology) antibody or an anti-GFP antibody (ab290, Abcam), which was capable of recognition of the YFP protein. The RNA/protein immunoprecipitation was treated with Proteinase K or SDS lysis buffer, respectively, for RNA purification and western blot.

**RNA extraction and quantitative RT-PCR**

Total RNA was isolated from the cells or tissues using RNAiso Plus Reagent (TaKaRa, 9109). A total of 500 ng of RNA was reverse transcribed to cDNA with the PrimeScript^TM^ RT reagent Kit (TaKaRa, RR037A). The obtained cDNA was diluted 1:10 with ddH_2_O and used for each qRT-PCR. Reactions were run on an Agilent Stratagene Mx3000 instrument using SYBR Premix Ex Taq^TM^ (TaKaRa, RR420A) or iTaqTM Universal SYBR Green Supermix (Bio-Rad, 1725125). The GAPDH or 18S gene was employed as an internal control. The relative expression level was calculated using the 2^-ΔΔCt^ method. The qRT-PCR primer sequences used in this study are listed in Supplementary Table S2.

**Western blot**

Cells were washed twice in cold PBS and incubated in 1×SDS lysis buffer with 1× PhosSTOP protease inhibitor (04693132001, Roche) for 15 min on ice. Equal amounts of protein were separated with SDS-PAGE gel and transferred to PVDF membranes (ISEQ00010, Millipore). The membranes were blocked with 3% BSA (#0617C258, Amresco, USA) for at least 1 h and incubated with antibodies against cleaved PARP (CY5035, Abways, Shanghai), cleaved caspase 3 (#9661, Cell Signaling Technology, Danvers, MA, USA), GAPDH (AP0063, Bioworld, Minnesota, USA), FUBP1-Rb (ab192867, Abcam, Cambridge, MA, USA), FUBP1-Ms (sc-271241, Santa Cruz Biotechnology, CA, USA), Tubulin (ap0064, Bioworld), Histone 3 (ab1791, Abcam), and flag-Ms (GNI4110-FG, GNI, Japan). After incubation with secondary antibodies, including HRP-Ms (#7074, Cell Signaling Technology) and HRP-Rb (#7076, Cell Signaling Technology), signals were visualized by enhanced chemiluminescence (ECL) (ImageQuant LAS 4000 mini).

**Cytoplasmic and nuclear fractionation**

Nuclear and cytoplasmic fractionation were performed as previously described^1^. Harvested cells were gently resuspended in 100 μL lysis buffer A (50 mM Tris-HCl pH 8.0, 50 mM NaCl, 1.5 mM MgCl_2_, and 0.5% NP40) for 5 min and then centrifuged at 1,000 g for 5 min. The supernatant contained the cytoplasmic fraction, added to 1 mL RNAiso Plus for cytoplasmic RNA purification or 100 μL RIPA (KGP701-100, Keygen Biotech, Jiangsu, China) for protein detection. Purified nuclear pellets washed with lysis buffer A were resuspended in 1 mL RNAiso Plus for nuclear RNA purification or 100 μL RIPA for protein detection. Cytoplasmic and nuclear RNA/protein detection were subsequently performed by qRT-PCR and western blot assays.

**Chromatin immunoprecipitation**

A total of 1×10^7^ cells were harvested and crosslinked with 37% formaldehyde for 10 min, followed by 1.25 M glycine quenching for 5 min at room temperature. Cell pellets were lysed and sonicated using an M220 Focused-ultrasonicator (Covaris) to generate 200-750 bp fragments. Immunoprecipitation was performed by rotating samples at 4℃ for at least 4 h with magnetic beads (ChIP-Grade Protein G beads; Cat# 9006s; Cell Signaling Technology) prebound to 3 μg of antibodies. The samples were reverse crosslinked overnight at 65°C overnight, treated with 1 μL of RNase A and 1 μL proteinase K (25 mg/mL). DNA was then extracted with phenol-chloroform (Sangon Biotech) and used for qRT-PCR analysis. The antibodies in ChIP assays were used as followed: anti-FUBP1 (sc-271241, Santa Cruz Biotechnology), anti-RNA Polymerase II (05-623, Millipore) and anti-IgG-Ms (12-371, Millipore, MA, USA). The fold enrichment was relative to the input DNA. The primers used in ChIP-qPCR are listed in Supplementary Table S3.

**Immunofluorescence staining**

For cell immunostaining, the cells were washed with PBS and fixed with 4% PFA at room temperature for 20 min. For tumor tissue immunostaining, the tissues were fixed with 4% PFA and dehydrated with 15% and 30% sucrose, respectively. The sections of tissues underwent antigen retrieval in solution (1.8 mM citrate acid and 8.2 mM trisodium citrate dihydrate, pH 6.0) at 97℃ for 20 min. The fixed cells or tissue sections were permeabilized with 0.1% Triton X-100 for 8 min and then blocked with 10% donkey serum for 1 h at room temperature; the samples were subsequently incubated with the appropriate dilution of primary antibodies (FUBP1, 1:100, sc-271241, Santa Cruz Biotechnology; cleaved caspase 3, 1:500, #9661, Cell Signaling Technology) overnight followed by incubation with an Alexa Fluor-488- or Alexa Fluor-594-conjugated secondary antibody (Invitrogen, A21206, A21203); then, the samples were counterstained with Hoechst 33342 at 4℃ for 1.5 h. Finally, the cells and sections were observed under a Nikon A1R confocal microscope (Nikon, Japan).

**DNA extraction and Bisulfite sequencing analysis**

Promoter CpG methylation was analyzed by bisulfite PCR as previously described^2^. CpG methylation of the NORAD promoter was analyzed at MethPrimer (http://www.urogene.org/cgi-bin/methprimer/methprimer.cgi). Briefly, genomic DNA of cells and tumor tissues (n=5, each group) was treated with RNase A (NEB) and extracted using a genomic DNA extraction kit (TIANGEN). A total of 1 μg of DNA was then modified by bisulfite treatment overnight and used for nested bisulfate-PCR. PCR products were cloned into the pMD19-T vector (TaKaRa) and then 10 clones of each group/patient were selected for sequencing. The BSP PCR primers are listed in Supplementary Table S5.

**TUNEL**

The cells and sections of tumor tissues were subjected to a one-step TUNEL Apoptosis Assay Kit (C1090, Beyotime, Shanghai, China) according to the manufacturer’s protocol. Briefly, cells and sections of tissues were fixed in 4% PFA for 30 min at room temperature, treated with 0.5% Triton X-100 for 5 min and labeled with 1 μL terminal deoxynucleotidyl transferase, 4 μL dilution buffer, and 45 μL fluorescein-12-dUTP dilution for each sample for 60 min at 37℃ in the dark. Cell nuclei were stained with Hoechst 33342. All fluorescent images were examined using a Nikon A1R confocal microscope (Nikon, Japan).

**Immunohistochemistry**

Paraffin-embedded sections of EC tumor tissues were deparaffinized and rehydrated, followed by antigen retrieval. After primary and secondary antibody (listed in immunofluorescence staining) incubation, the slides were finally incubated with diaminobenzidine (DAB) (Dako, USA) and counterstained with hematoxylin (Sigma Chemical Co, USA).

**TCGA analysis**

The TCGA-UCEC datasets (544 EC specimens and 23 normal tissues) for the expression levels of NORAD and FUBP1 were downloaded (https://tcga-data.nci.nih.gov/tcga/). The clinical features of patients, including the survival time and survival status, were collected based on the patient records. The significance threshold of the differential expression was determined based on the p-value (<0.05) upon t-test. A log-rank test was performed to predict the prognostic value of NORAD.

**GSEA**

Enrichment analyses for custom signatures using gene sets associated with FUBP1 expression in liver cancer from the TCGA dataset were carried out using the non-parametric GSEA software with all default settings. The statistical significance was assessed by comparing the enrichment score.

**PDX model and histological analysis**

Fresh EC tumor tissues from surgeries were dissected into 2.5×2.5×2.5 mm pieces, mixed with 20% Matrigel (Corning, Tewksbury, MA, USA) and subcutaneously injected into the flanks of 9 week-old female NCG mice (NOD-Prkdc^em26Cd52^Il2rg^em26Cd22^/ NjuCrl) (GemPharmatech, Jiangsu, China). After 12 weeks of injection, the tumor size reached more than 500 mm^3^, and the tumors were dissected and re-injected to expand the tumor tissue. The EC PDX model was successfully established, verified by paraffin-embedded tumor tissues and subsequently stained by hematoxylin and eosin (H&E). We used 5 mice from each group for Aza treatment. When the tumor reached 50 mm^3^, the mice were treated with 2.5 mg/kg Aza (S1782, Selleck) via intraperitoneal injection. The mice were dosed for five days a week with a two-day rest, lasting four cycles and including a rest period of one week between cycles 2 and 3. At 28 days post-treatment, the mice were euthanized and analyzed. These studies were approved by the Institutional Animal Care and Use Committee (no. TJLAC-019-103).

**Supplementary references**

1. Hwang, H. W., Wentzel, E. A. & Mendell, J. T. A hexanucleotide element directs microRNA nuclear import. *Science* **315**, 97-100 (2007).

2. Koh, K. P. et al. Tet1 and Tet2 regulate 5-hydroxymethylcytosine production and cell lineage specification in mouse embryonic stem cells. *Cell Stem Cell* **8**, 200-213 (2011).
